# Approximate, not Perfect Synchrony Maximizes the Downstream Effectiveness of Excitatory Neuronal Ensembles

**DOI:** 10.1186/2190-8567-4-10

**Published:** 2014-04-25

**Authors:** Christoph Börgers, Jie Li, Nancy Kopell

**Affiliations:** Department of Mathematics, Tufts University, Medford, MA 02155 USA; Department of Mathematics and Statistics, Boston University, Boston, MA 02215 USA

**Keywords:** Function of synchrony, Leakiness, Coincidence detection

## Abstract

The most basic functional role commonly ascribed to synchrony in the brain is that of amplifying excitatory neuronal signals. The reasoning is straightforward: When positive charge is injected into a leaky target neuron over a time window of positive duration, some of it will have time to leak back out before an action potential is triggered in the target, and it will in that sense be wasted. If the goal is to elicit a firing response in the target using as little charge as possible, it seems best to deliver the charge all at once, i.e., in perfect synchrony. In this article, we show that this reasoning is correct only if one assumes that the input ceases when the target crosses the firing threshold, but before it actually fires. If the input ceases later—for instance, in response to a feedback signal triggered by the firing of the target—the “most economical” way of delivering input (the way that requires the least total amount of input) is no longer precisely synchronous, but merely approximately so. If the target is a heterogeneous network, as it always is in the brain, then ceasing the input “when the target crosses the firing threshold” is not an option, because there is no single moment when the firing threshold is crossed. In this sense, precise synchrony is never optimal in the brain.

**Electronic supplementary material:**

The online version of this article (doi:10.1186/2190-8567-4-10) contains supplementary material, which is available to authorized users.

## 1 Introduction

Synchronization of neuronal firing is widely thought to be important in brain function. Synchrony and rhythms have been hypothesized to play roles, for instance, in directing information flow [1–3], binding the activity of different neuronal assemblies [4], protecting signals from distractors [5], enhancing input sensitivity [6, 7], and enhancing the downstream effectiveness of neuronal signals [8–10].

The case is simplest and strongest for the last of these hypothesized functional roles of synchrony: By synchronizing, an ensemble of excitatory neurons can amplify its downstream effect. In fact, when positive charge is injected into a leaky target neuron over a time window of positive duration, some of it will have time to leak back out before an action potential is triggered in the target, and it will in that sense be wasted. If the goal is to elicit a firing response in the target using as little charge as possible, it seems best to deliver the charge all at once, i.e., in perfect synchrony. Leaky neurons are often said to be *coincidence detectors* for this reason. This reasoning is commonplace and widely accepted in neuroscience. However, we show that whether or not it is actually correct depends on how one makes it precise; with one formalization that seems particularly natural to us, it is incorrect.

**Fig. 1:**
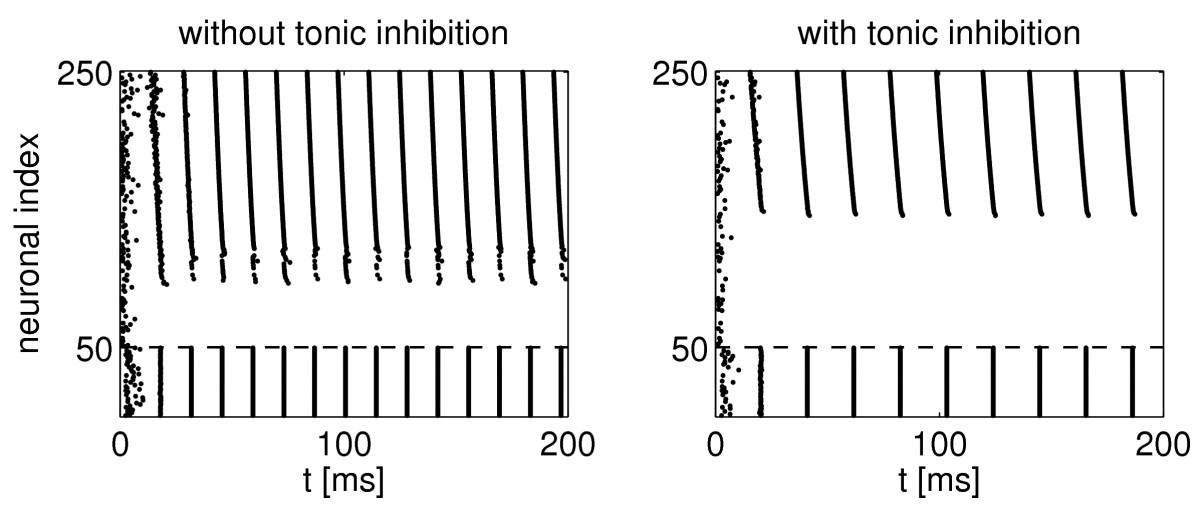
E-I networks without (*left*) and with (*right*) tonic inhibition of the E-cells. The tonic inhibition reduces the number of E-cells participating from 145 to 111. At the same time, the frequency of the rhythm drops from 61 Hz to 47 Hz, and the time that it takes for the first 100 E-cells to fire on each spike volley rises from about 2.9 ms to about 4.4 ms

Network simulations of the kind shown in Fig. 1 have motivated this study. The figure shows spike rastergrams of networks of excitatory and inhibitory neurons (E- and I-cells); see Sect. 2.6 for the complete details. There are 200 E-cells (above the dashed line in the figure) and 50 I-cells (below the dashed line). The E-cells receive a strong external drive, linearly graded in strength; cells with greater neuronal index receive a greater drive. The I-cells are driven weakly, and they fire in response to the E-cells only. The synaptic interaction of the E- and I-cells with each other creates a rhythm in the gamma frequency range (30–80 Hz). The frequency comes from the decay time constant of inhibition, which is assumed here to be 9 ms, reminiscent of GABA_A_-receptor-mediated inhibitory synapses [11]. The period of a 40-Hz rhythm, for example, is 25 ms, approximately the time it takes the inhibition to decay by a factor of 10 if the decay time constant is 9 ms. Rhythms of this sort are called PING (Pyramidal-Interneuronal Network Gamma) rhythms [12]. In the right panel of the figure, tonic inhibition, i.e., synaptic inhibition with a constant conductance, has been added to the E-cells. The result is a slower rhythm, with looser synchronization among the E-cells, and participation of fewer E-cells. Notice that fewer E-cells are needed to prompt the I-cell response, in spite of the fact that the E-cells are less tightly synchronized than in the left panel. In this sense, the less tightly synchronized E-cells in the right panel seem to be more effective than the more tightly synchronized ones in the left panel, which appears to contradict the idea that for excitatory synaptic transmission, greater synchrony results in greater effectiveness.

We emphasize that this paper is not about rhythms; Fig. 1 is merely a motivating example. Here we focus on a *single* excitatory spike volley, and we study how it triggers a firing response in a target neuron. In the examples in Fig. 1, the target neurons are the I-cells. Specifically, we study the effect of tighter or looser synchrony within a single excitatory spike volley.

The resolution of the puzzle raised by Fig. 1 lies in the observation that there are (at least) two fundamentally different ways of asking the question “Does synchrony maximize the effectiveness of ensembles of excitatory neurons?”, and they lead to different answers. Simplifying a bit, we can state the following two principles.

1. If the excitatory input is allowed to have the “foresight” of turning off as soon as the target crosses the firing threshold, i.e., as soon as firing becomes inevitable even without further input, then precise synchrony is indeed optimal, as the commonplace reasoning would suggest.
2. On the other hand, if the excitatory input lasts until the target actually fires (perhaps terminated by a feedback signal), then approximate, often quite imperfect synchrony is optimal.

Both principles can be made precise, proved, and computationally supported in numerous different ways. We will give examples of that in this article. However, intuitively the reasoning is very simple: When the input is made more synchronous, it becomes more effective at eliciting a firing response in the target, but more of it is wasted because it arrives between the time when the firing threshold is reached in the target and the time when the input turns off.

The central distinction that we draw in this paper is between maintaining the input until the target reaches its firing threshold, and maintaining the input until the target actually fires. Assuming that the input continues until the target reaches threshold, greater synchrony is more economical. However, assuming that the input continues until the target fires, or even longer, for instance until a feedback signal from the target arrives, there is an “optimally economical” degree of synchrony that is not perfect, and that can be quite far from perfect.

The E-to-I interaction in PING is an example of an excitatory signal terminated by a feedback signal from the target: The E-cells stop firing when the I-cells respond, and thereby they shut them off. In PING, therefore, approximate synchrony of the E-cells is “optimal” in the sense that the rhythm is maintained with the smallest number of E-cells firing.

There is little evidence of *perfect* synchrony in the brain. If synchrony is really functionally important, this begs the question why evolution did such a poor job perfecting it. Perhaps the arguments given in this article point towards an answer: Making our terms precise in one possible and, we think, very natural way, we find that imperfect synchrony is more “economical” than perfect synchrony.

## 2 Models

In this section we introduce the model target neurons that we will use throughout the paper. For completeness, we also specify the details of the network of Fig. 1.

We frequently use linear integrate-and-fire neurons in this paper, since analysis is easiest for them. For greater biophysical realism, we also use simple (single-compartment) Hodgkin–Huxley-like model neurons, for which we report numerical results, but no analysis. The theta neuron is in between: It is still simple enough for the sort of analysis that we are interested in here, but it is more realistic than the linear integrate-and-fire neuron.

### 2.1 Linear Integrate-and-Fire Model

In the linear integrate-and-fire (LIF) neuron, we take the membrane potential, *v*, to be scaled and shifted so that the firing threshold is 1, and the reset voltage is 0. The model then becomes

1 $$\frac{dv}{dt}=-\frac{v}{\tau }+I\;\text{if }v<1,$$

2 v(t+0)=0if v(t−0)=1,

where v(t−0) and v(t+0) denote left- and right-sided limits, respectively, τ>0 is the membrane time constant, and *I* is normalized external drive. Although the normalized membrane potential *v* is non-dimensional, we find it convenient to think of *t* and *τ* as quantities with the physical dimension of time, measured in ms. As a result, *I* is a reciprocal time.

Among other things, we will study how a brief positive input pulse elicits an action potential. In this context, *I* will be a continuous function of *t*, about which we assume

$$I(t)\geq 0\;\text{for all }t\geq 0,\;\;\lim_{t\to \infty }I(t)=0,\;\text{and}\;0<q=\int_{0}^{\infty }I(t)dt<\infty .$$

We interpret *q* as the (normalized) total charge injected into the neuron. We assume that I(t) is of significant size for t≤1, but not for t≫1. (For numerical illustrations, we will use $I(t)=rte^{-t}$, with r>0.) Thus the “duration” of the input pulse *I* is on the order of 1 ms.

We will discuss in what sense there is coincidence detection, i.e., in what sense more rapid delivery of excitatory input is more effective. For this purpose, we consider

$$I_{\varepsilon }(t)=\frac{1}{\varepsilon }I\left(\frac{t}{\varepsilon }\right),\;\varepsilon >0.$$

For all ε>0, the total amount of charge injected is

$$\int_{0}^{\infty }I_{\varepsilon }(t)dt=\int_{0}^{\infty }\frac{1}{\varepsilon }I\left(\frac{t}{\varepsilon }\right)=\int_{0}^{\infty }I(t)dt=q.$$

Note that $I_{\varepsilon }$ is of significant size for t≤ε, but not for t≫ε. Thus the duration of the input pulse $I_{\varepsilon }$ is on the order of *ε* (time measured in ms). For smaller *ε*, the same amount of charge is delivered in a briefer time period; this is why we think of smaller *ε* as modeling greater synchrony of inputs. As ε→0, $I_{\varepsilon }$ converges to qδ(t), where *δ* denotes the Dirac delta function at t=0. In this limit, the effect of the input pulse $I_{\varepsilon }$ becomes an instantaneous increase in the membrane potential by *q*.

To clarify in which sense smaller *ε* corresponds to more synchronous input, think of I(t) as being approximated by a sum of *δ*-functions:

3 $$I(t)\approx \sum_{j=1}^{\infty }w_{j}\delta \left(t-(j-1/2)\Delta \right)$$

with

4 $$w_{j}=\int_{(j-1)\Delta }^{j\Delta }I(s)ds,$$

where Δ>0 is small. Physically, this amounts to approximating the input current *I* by a sequence of weak instantaneous charge injections, arriving at times (j−1/2)Δ, j=1,2,3,… . These instantaneous charge injections can be understood as models of very fast, i.e., very rapidly decaying excitatory synaptic inputs. (Technically, the right-hand side of (3) converges to I(t) as Δ→0 in the distributional sense.) The input pulse $I_{\varepsilon }$ can be approximated by

5 $$I_{\varepsilon }(t)\approx \sum_{j=1}^{\infty }w_{j}\delta \left(t-(j-1/2)\varepsilon \Delta \right),$$

with the same weights $w_{j}$. (Again, technically the right-hand side converges to the left-hand side in the distributional sense as Δ→0.) Thus $I_{\varepsilon }$ is approximated by the same sequence of weak input pulses as *I*, but the time between subsequent input pulse arrivals is *ε* Δ instead of Δ; that is, the input pulses arrive more synchronously when *ε* is smaller.

To better understand coincidence detection, we will examine how the solution of

6 $$\frac{dv_{\varepsilon }}{dt}=-\frac{v_{\varepsilon }}{\tau }+I_{\varepsilon }(t),\;v_{\varepsilon }(0)=0,$$

depends on *ε*. When ε=1, we write *v* instead of $v_{\varepsilon }=v_{1}$. To make the dependence on *τ* explicit, we sometimes write $v_{\varepsilon }(t,\tau )$ or v(t,τ); even when using this notation, however, we usually denote the derivative with respect to *t* by d/dt, not ∂/∂t. Since I(t)≥0, it is guaranteed that $v_{\varepsilon }(t,\tau )\geq 0$ for all ε>0, t≥0, and τ>0. We use the notation

7 $$M_{\varepsilon }=\max_{t\geq 0}v_{\varepsilon }(t).$$

An action potential is elicited by the input $I_{\varepsilon }$ if and only if $M_{\varepsilon }\geq 1$.

### 2.2 Theta Model

The theta neuron, first proposed by Ermentrout and Kopell [13], is equivalent to a specific form of the quadratic integrate-and-fire (QIF) model. The equation governing the membrane potential *v* is now

8 $$\frac{dv}{dt}=-\frac{v}{\tau }(1-v)+I.$$

For I<1/(4τ), Eq. (8) has two fixed points, $v_{-}$ and $v_{+}$, with

9 $$v_{\pm }=\frac{1}{2}\pm \sqrt{\frac{1}{4}-\tau I}.$$

The fixed point $v_{-}$ is stable and $v_{+}$ is unstable. The two fixed points collide and annihilate each other in a saddle-node bifurcation as *I* rises above 1/(4τ).

The quadratic nature of the right-hand side of (8) has the effect that *v* rises from 1 to +∞ and from −∞ to 0 in a finite (and brief) amount of time. One obtains a simplified model by moving the firing threshold to +∞, and the reset voltage to −∞:

10 v(t+0)=−∞if v(t−0)=∞.

With the change of coordinates

11 $$v=\frac{1}{2}\left(1+\tan\frac{\theta }{2}\right),$$

the model then becomes

12 $$\frac{d\theta }{dt}=-\frac{\cos\theta }{\tau }+2I(1+\cos\theta ).$$

When I<1/(4τ), there are two fixed points, corresponding to the two fixed points of the QIF neuron given in (9):

13 $$\theta_{\pm }=\pm \arccos\frac{2\tau I}{1-2\tau I}.$$

The fixed point $\theta_{-}$ is stable and $\theta_{+}$ is unstable. When we refer to the *theta model*, we mean (12) or, equivalently, (8) and (10). To *fire* means to reach θ=π mod 2π, or equivalently, v=∞. Ermentrout and Kopell [13] used τ=1/2.

We will study how a brief positive input pulse into a theta neuron elicits an action potential, using the same setup as in Sect. 2.1. Equation (6) becomes

14 $$\frac{dv_{\varepsilon }}{dt}=-\frac{v_{\varepsilon }}{\tau }(1-v_{\varepsilon })+I_{\varepsilon },\;v_{\varepsilon }(0)=0.$$

(Note that v=0 is the stable equilibrium of Eq. (8) when I=0.) As in Sect. 2.1, we sometimes write $v_{\varepsilon }(t,\tau )$ to make the dependence on *τ* explicit, and we skip the subscript *ε* when ε=1. Also as in Sect. 2.1, we note that $v_{\varepsilon }(t,\tau )\geq 0$ for all ε>0, t≥0, and τ>0.

The definition of $M_{\varepsilon }$ (compare Eq. (7)) must be modified slightly here:

15 $$M_{\varepsilon }=\{\begin{array}{cc} \sup_{t\geq 0}v_{\varepsilon }(t) & \text{if }v_{\varepsilon }(t)\text{ remains finite for all }t\geq 0,\\ \infty & \text{if }v_{\varepsilon }\text{ becomes infinite in finite time}. \end{array}$$

An action potential is elicited by the input pulse $I_{\varepsilon }$ if and only if $M_{\varepsilon }>1$. We note that $M_{\varepsilon }>1$ is equivalent to $M_{\varepsilon }=\infty$, since $v_{\varepsilon }$ will reach ∞ in finite time as soon as it exceeds 1.

### 2.3 Wang–Buzsáki Model

The well-known Wang–Buzsáki (WB) neuron [14] is patterned after fast-firing interneurons in rat hippocampus. The ionic currents are those of the classical Hodgkin–Huxley neuron, i.e., spike-generating sodium, delayed rectifier potassium, and leak currents; we refer to [14] or [[11], Appendix 1] for all details.

### 2.4 A Rapid Volley of Excitatory Synaptic Inputs into a Single Target Neuron

Throughout most of this paper, we will think about a single target neuron driven by input. This input may be a current pulse, for instance of the form $I_{\varepsilon }$ described earlier, or more realistically, a sequence of weak excitatory synaptic input pulses, modeled by a term of the form

16 $$\bar{g}s(t)(v_{\mathrm{rev},e}-v)$$

on the right-hand side of the equation governing the evolution of *v*. Here $v_{\mathrm{rev},e}$ is the *synaptic reversal potential*. For the WB model, we use $v_{\mathrm{rev},e}=0$, following [[11], Appendix 1]. For the LIF and theta models, we use $v_{\mathrm{rev},e}=5$. As in a real neuron, this is the threshold voltage (v=1) plus several times (namely, four times) the difference between threshold (v=1) and reset (v=0). The *synaptic gating variable*s(t) will be assumed to jump upwards periodically with period Δ>0 (time measured in ms):

s(t+0)=s(t−0)+1if t=Δ,2Δ,3Δ,….

The variable *s* should be thought of as the sum of gating variables associated with multiple different weak synapses, with an accumulating effect far from saturation; this is why *s* is not assumed to be bounded by 1. For simplicity, the time Δ between the arrival of input pulses is assumed to be constant. The factor $\bar{g}$ in (16) represents the maximal conductance (or conductance density) of one of the weak synapses, and it will be taken to be small. Between jumps, we assume *s* to decay with time constant 3 ms:

17 $$\frac{ds}{dt}=-\frac{s}{3}.$$

The decay time constant of 3 ms is chosen to mimic AMPA-receptor-mediated synapses [11].

### 2.5 Reduced Traub–Miles Model

In our network model, the inhibitory cells are WB neurons, and the excitatory ones reduced Traub–Miles (RTM) neurons. The RTM model is due to Ermentrout and Kopell [15], patterned after a more complicated, multi-compartment model of Traub and Miles [16], and it is used here in the form stated in detail in [[11], Appendix 1]. It is a single-compartment model of a pyramidal (excitatory) cell in rat hippocampus. As for the WB neuron, the ionic currents are those of the classical Hodgkin–Huxley neuron, i.e., spike-generating sodium, delayed rectifier potassium, and leak currents.

### 2.6 Network Model

The only network simulation in this paper is the motivating example shown in Fig. 1. The model network consists of 200 RTM neurons (E-cells) and 50 WB neurons (I-cells). There is all-to-all synaptic connectivity, modeled as described in [[11], Appendix 1], with no gap junctions. The following parameter values specify the network in the left panel of the figure completely (see [[11], Appendix 1]):

- The drive to the *j* th E-cell (strictly speaking, drive density, measured in μA/cm^2^) is $I_{E,j}=1.5+j/200$, 1≤j≤200. (The *j* th E-cell is labeled 50+j in Fig. 1, because the 50 I-cells are labeled first.) The drives to the I-cells are zero. There is no stochastic drive here.
- The total synaptic conductances (strictly speaking, conductance densities, measured in mS/cm^2^) are ${\hat{g}}_{EI}=0.4$, ${\hat{g}}_{IE}=1$, ${\hat{g}}_{II}=0.6$, and ${\hat{g}}_{EE}=0$. The conductance associated with a single I→E-synapse, for instance, is ${\hat{g}}_{IE}/50=0.02$.
- The reversal potentials (measured in mV) of the excitatory and inhibitory synapses are $v_{\mathrm{rev},E}=0$ and $v_{\mathrm{rev},I}=-67$.
- The rise and decay time constants of synaptic inhibition (measured in ms) are $\tau_{R,E}=0.1$, $\tau_{D,E}=3$, $\tau_{R,I}=0.3$, and $\tau_{D,I}=9$.

In the right panel of the figure, the extra term $0.2(-67-v_{j})$ is added to the right-hand side of the equation governing the membrane potential $v_{j}$ of the *j* th E-cell, 1≤j≤200, to model tonic inhibition affecting the E-cells.

### 2.7 Computer Codes

Each figure in this paper is generated by a single, stand-alone Matlab program. All of these programs can be obtained by e-mail from the first author.

## 3 If the Excitatory Signal Ceases when the Target Crosses the Firing Threshold, Synchrony Is Optimally Efficient

We will give several settings in which the above statement can be made rigorous. Here the target is always a single neuron, not a network. In Fig. 1, the target of the excitatory input volleys is a network, namely the ensemble of I-cells. However, they are synchronized so tightly that we might as well assume a single I-cell. For a comment on the case when the target is itself a network that is not perfectly synchronous, see the Discussion.

### 3.1 Constant Current Input Driving a LIF Neuron

This is the most commonplace version of the argument. Consider a LIF neuron driven by a constant input 1/Δ, with Δ>0:

18 $$\frac{dv}{dt}=-\frac{v}{\tau }+\frac{1}{\Delta },\;v(0)=0.$$

We think of Δ as the time between the individual pulses of a rapid input volley, as in Sect. 2.4. In (18) we simplify by equating the frequency of input pulses within the volley, 1/Δ, with the strength of a constant input current. Smaller Δ, i.e., larger input, should be thought of as modeling more synchronous input from multiple sources.

An action potential occurs if and only if Δ<τ, and in that case it occurs at time

19 $$T_{\Delta }=\tau \ln\frac{\tau /\Delta }{\tau /\Delta -1}.$$

Note that the time of firing is, in this model, the same as the time at which the firing threshold is reached. An action potential occurs instantly (by definition) when *v* reaches 1. If the input ceased before *v* reaches 1, no action potential would occur. The total charge needed to elicit an action potential is

$$Q_{\Delta }=\text{time}\times \text{input current}=T_{\Delta }\times \frac{1}{\Delta }=\frac{T_{\Delta }}{\Delta }.$$

Using (19), we find

20 $$Q_{\Delta }=\varphi \left(\frac{\tau }{\Delta }\right)\;\text{with }\varphi (s)=s\ln\frac{s}{s-1}.$$

**Fig. 2:**
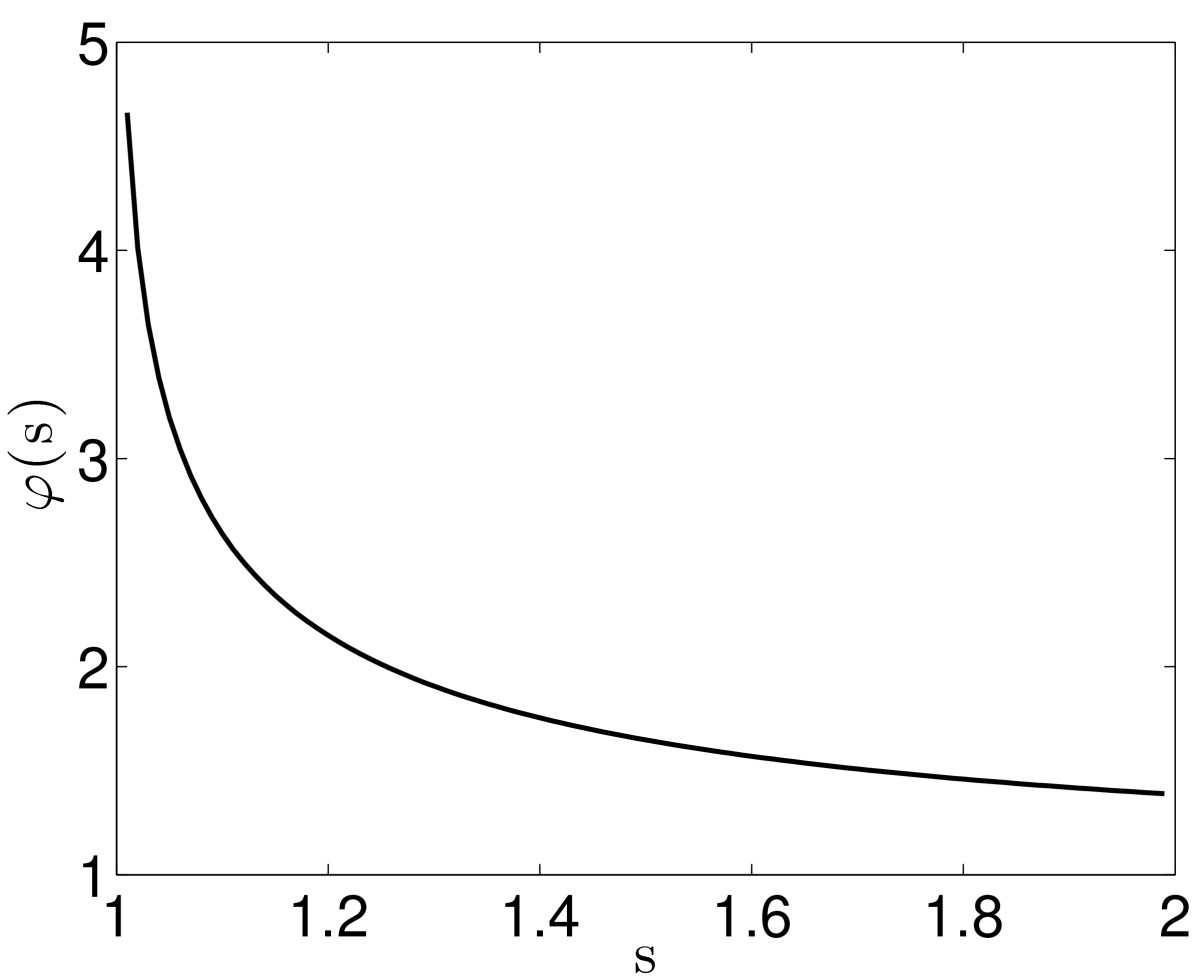
The function *φ* in Eq. (20)

Figure 2 shows the graph of the function *φ*. It is strictly decreasing, so $Q_{\Delta }$ is a strictly increasing function of Δ. More synchronous input (smaller Δ) produces an action potential in the target more efficiently (smaller $Q_{\Delta }$). The fact that $Q_{\Delta }$ is a function of τ/Δ (not of Δ alone) reflects that leakiness is what matters here.

### 3.2 Current Input Pulse of General Shape Driving a LIF Neuron

We turn to a second way of making precise the notion that excitatory current input becomes more effective when delivered more synchronously. Consider a linear integrate-and-fire neuron subject to a positive current pulse, as described in Sect. 2.1, where the notation used here was introduced. The issue of coincidence detection is linked to leakiness, and we therefore first think about how *v* depends on *τ*.

**Lemma 1** (a) *Let*$0<\tau_{1}<\tau_{2}$. *Then for all*t≥0, $v(t,\tau_{1})\leq v(t,\tau_{2})$. *Furthermore*, *if*$v(t,\tau_{2})>0$, *then*$v(t,\tau_{1})<v(t,\tau_{2})$. (b) $\lim_{\tau \to 0}\max_{t\geq 0}v(t,\tau )=0$.

*Proof* (a) By standard theory of ordinary differential equations, $v(t,\tau_{1})\leq v(t,\tau_{2})$ for all t≥0 because the right-hand side of the differential equation (1) is an increasing function of *τ*. We will show now that the inequality is strict if $v(t,\tau_{2})>0$. Suppose that on the contrary, $t_{*}>0$ with $v(t_{*},\tau_{1})=v(t_{*},\tau_{2})=v_{*}>0$. Then

$$\frac{dv}{dt}(t_{*},\tau_{1})=-\frac{v_{*}}{\tau_{1}}+I(t_{*})<-\frac{v_{*}}{\tau_{2}}+I(t_{*})=\frac{dv}{dt}(t_{*},\tau_{2}),$$

and therefore $v(t,\tau_{1})<v(t,\tau_{2})$ for $t<t_{*}$, $t_{*}-t$ sufficiently small. However, we already know that this is impossible. (b) Using variation of the constant, we find

$$v(t,\tau )=\int_{0}^{t}e^{-(t-s)/\tau }I(s)ds.$$

This implies part (b) of the lemma. □

**Lemma 2**$M_{\varepsilon }$*is a strictly decreasing function of*ε>0*with*$\lim_{\varepsilon \to 0}M_{\varepsilon }=q$*and*$\lim_{\varepsilon \to \infty }M_{\varepsilon }=0$.

*Proof* We will first show that

21 $$v_{\varepsilon }(t,\tau )=v\left(\frac{t}{\varepsilon },\frac{\tau }{\varepsilon }\right).$$

To verify (21), we first note that both sides of (21) are zero at t=0. We next carry out a brief calculation to show that the right-hand side solves the differential equation in (6), which the left-hand side solves by definition. In this calculation, we will use the notation $v_{t}$ for the partial derivative of v=v(t,τ) with respect to *t*. With this notation,

$$\frac{d}{dt}\left(v\left(\frac{t}{\varepsilon },\frac{\tau }{\varepsilon }\right)\right)=\frac{1}{\varepsilon }v_{t}\left(\frac{t}{\varepsilon },\frac{\tau }{\varepsilon }\right)$$

by the chain rule,

$$\frac{1}{\varepsilon }v_{t}\left(\frac{t}{\varepsilon },\frac{\tau }{\varepsilon }\right)=\frac{1}{\varepsilon }\left[-\frac{1}{\tau /\varepsilon }v\left(\frac{t}{\varepsilon },\frac{\tau }{\varepsilon }\right)+I\left(\frac{t}{\varepsilon }\right)\right]$$

because *v* satisfies (1) (the “*τ*” in (1) is replaced by τ/ε here, and the time at which we evaluate both sides of (1) is t/ε), and

$$\frac{1}{\varepsilon }\left[-\frac{1}{\tau /\varepsilon }v\left(\frac{t}{\varepsilon },\frac{\tau }{\varepsilon }\right)+I\left(\frac{t}{\varepsilon }\right)\right]=-\frac{1}{\tau }v\left(\frac{t}{\varepsilon },\frac{\tau }{\varepsilon }\right)+I_{\varepsilon }(t)$$

by the definition of $I_{\varepsilon }$. This concludes the derivation of (21).

Equation (21) implies

22 $$M_{\varepsilon }=\max_{t\geq 0}v_{\varepsilon }(t,\tau )=\max_{t\geq 0}v\left(\frac{t}{\varepsilon },\frac{\tau }{\varepsilon }\right)=\max_{t\geq 0}v\left(t,\frac{\tau }{\varepsilon }\right).$$

Part (a) of Lemma 1 now implies that $M_{\varepsilon }$ is a strictly decreasing function of *ε*.

We pointed out in Sect. 2.1 that in the limit as ε→0, $I_{\varepsilon }$ approaches qδ(t). Thus in this limit, $v_{\varepsilon }$ jumps from 0 to *q* at time t=0, then decays. This implies $M_{\varepsilon }\to q$ as ε→0. Part (b) of Lemma 1, combined with (22), implies $M_{\varepsilon }\to 0$ as ε→∞. □

**Fig. 3:**
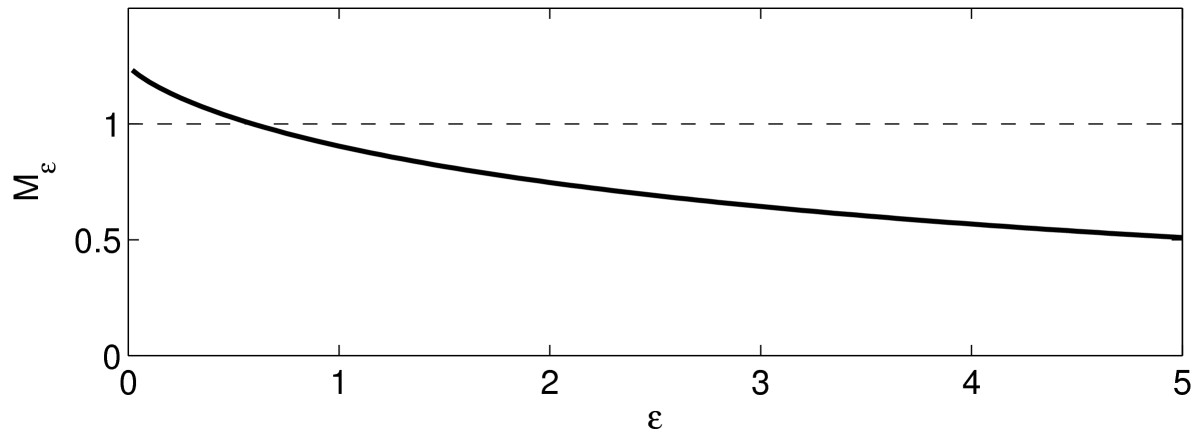
$M_{\varepsilon }$ as a function of *ε*, for the LIF neuron with τ=10, $I(t)=1.25te^{-t}$

Figure 3 illustrates the statement of Lemma 2 by showing the graph of $M_{\varepsilon }$, as a function of *ε*, for τ=10, $I(t)=1.25te^{-t}$.

**Theorem 1***If*q>1, *there exists an*$\varepsilon_{0}>0$*such that*$I_{\varepsilon }$*elicits an action potential for*$0<\varepsilon \leq \varepsilon_{0}$, *but not for*$\varepsilon >\varepsilon_{0}$. *If*q≤1, *then*$I_{\varepsilon }$*does not elicit an action potential for any*ε>0.

*Proof* This immediately follows from Lemma 2. □

The theorem shows that input becomes more effective when delivered more rapidly: If a given pulse succeeds at eliciting an action potential, then the same pulse, delivered faster, will succeed as well.

### 3.3 Current Input Pulse of General Shape Driving a Theta Neuron

We repeat the analysis of the preceding section for a target modeled as a theta neuron. So we now consider a theta neuron, written in terms of *v*, subject to a positive current pulse; see Sect. 2.2. As in Sect. 3.2, we begin by analyzing the effect of leakiness on the membrane potential.

**Lemma 3** (a) *Let*$0<\tau_{1}<\tau_{2}$. *Let*T>0*be chosen so that*$v(t,\tau_{2})<1$*for*t∈[0,T]. *Then for all*t∈[0,T], $v(t,\tau_{1})\leq v(t,\tau_{2})$. *Furthermore*, *if*$v(t,\tau_{2})>0$, *then*$v(t,\tau_{1})<v(t,\tau_{2})$. (b) $\lim_{\tau \to 0}\sup_{t\geq 0}v(t,\tau )=0$.

*Proof* (a) Same as proof of Lemma 1. (b) Let $S=\max_{t\geq 0}I(t)$, and assume that *τ* is so small that τS<1/4. Then

23 $$-\frac{v}{\tau }(1-v)+S<0,$$

for

$$\frac{1}{2}-\sqrt{\frac{1}{4}-\tau S}<v<\frac{1}{2}+\sqrt{\frac{1}{4}-\tau S}.$$

Note that (23) implies

$$-\frac{v}{\tau }(1-v)+I(t)<0.$$

Consequently the solution *v* of

$$\frac{dv}{dt}=-\frac{v}{\tau }(1-v)+I(t),\;v(0)=0$$

cannot exceed $1/2-\sqrt{1/4-\tau S}$. This bound converges to 0 as τ→0, implying (b). □

**Lemma 4***As long as*$M_{\varepsilon }$*is less than* 1, *it is a strictly decreasing function of*ε>0, *and*$M_{\varepsilon }\to 0$*as*ε→∞.

*Proof* Same as proof of Lemma 2. □

**Fig. 4:**
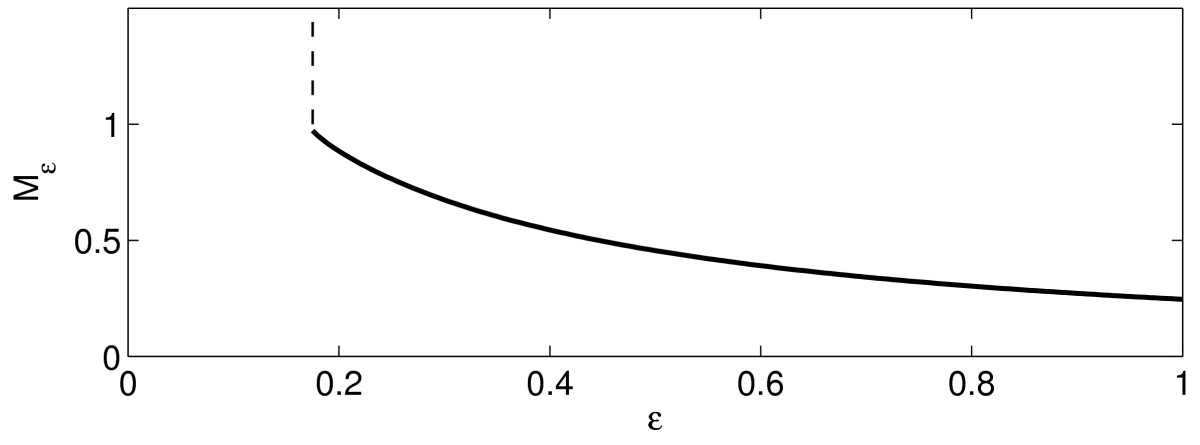
$M_{\varepsilon }$ as a function of *ε*, for the theta neuron with τ=1/2, $I(t)=1.25te^{-t}$

Figure 4 illustrates the statement of Lemma 4 by showing the graph of $M_{\varepsilon }$, as a function of *ε*, for τ=1/2, $I(t)=1.25te^{-t}$.

**Theorem 2***If*q>1, *there exists an*$\varepsilon_{0}>0$*such that*$M_{\varepsilon }=\infty$*for*$\varepsilon <\varepsilon_{0}$, $M_{\varepsilon_{0}}=1$, *and*$M_{\varepsilon_{0}}<1$*for*$\varepsilon >\varepsilon_{0}$. *If*q≤1, *then*$M_{\varepsilon }<1$*for all*ε>0.

*Proof* This follows immediately from Lemma 4. □

Again we see that input becomes more effective when delivered more rapidly: If a given pulse succeeds at eliciting an action potential, then the same pulse, delivered faster, will succeed as well.

We conclude this subsection with a tangential comment. When $\varepsilon <\varepsilon_{0}$, $v_{\varepsilon }$ stays below 1 for all times, and converges to 0 as t→∞. When $\varepsilon >\varepsilon_{0}$, $v_{\varepsilon }$ rises above 1 at a finite time. What is the behavior of $v_{\varepsilon_{0}}(t)$? It can be shown that $v_{\varepsilon_{0}}(t)\to 1$ as t→∞; we omit the proof of this result because it is not central to what this article is about. The result sounds surprising at first, since $I_{\varepsilon_{0}}(t)\to 0$ as t→∞ and 1 is a *repelling* fixed point of the equation

$$\frac{dv_{\varepsilon_{0}}}{dt}=-\frac{v_{\varepsilon_{0}}}{\tau }(1-v_{\varepsilon_{0}}).$$

**Fig. 5:**
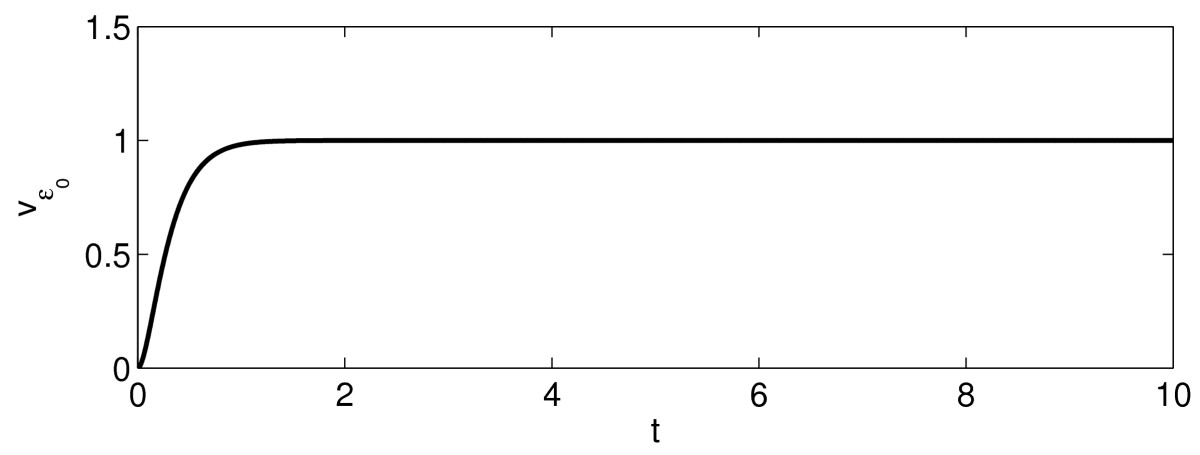
Convergence to a repelling fixed point resulting from just the right input pulse: $v_{\varepsilon_{0}}(t)$ converges to 1 as t→∞

However, the repulsion is overcome by the positive input. Figure 5 illustrates this point for τ=1/2, $I(t)=1.25te^{-t}$.

### 3.4 Sequence of Weak Instantaneous Positive Charge Injections Driving a LIF Neuron

The analyses given so far assume continuous current inputs. Of course, in the brain, inputs come as synaptic pulses. The simplest model involving a sequence of weak input pulses, not a continuous current input, is

$$\frac{dv}{dt}=-\frac{v}{\tau }+w\sum_{k=1}^{\infty }\delta (t-k\Delta ),\;v(0)=0,$$

where *δ* denotes, as before, the Dirac delta function, w∈(0,1), and Δ>0. It is straightforward to verify that *v* will reach the threshold 1 eventually if and only if

$$w>1-e^{-\Delta /\tau },$$

and that the number $N_{\Delta }$ of input pulses required to make *v* reach 1 decreases as Δ decreases. We omit the derivation of this unsurprising result.

### 3.5 Sequence of Weak Excitatory Synaptic Pulses Driving a WB Neuron

We now give our final and most realistic illustration of the principle that synchrony makes excitatory input into a target neuron optimally efficient, provided that the input is allowed to cease when the target crosses the firing threshold.

**Fig. 6:**
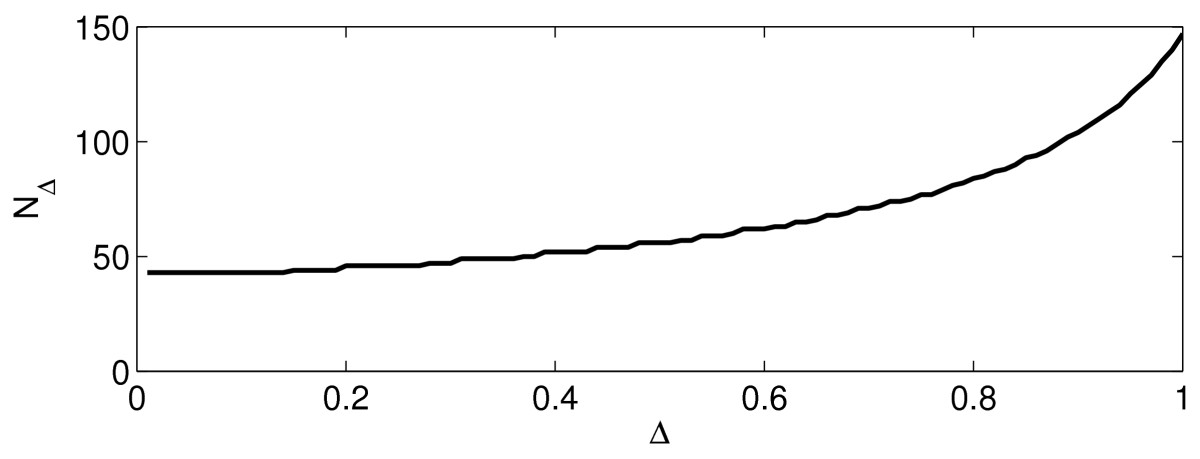
The number, $N_{\Delta }$, of synaptic input pulses required to trigger an action potential in a WB neuron, as a function of Δ=time between pulses

We examine a WB neuron with zero external drive, resting at its stable fixed point at time zero, and then subject to weak excitatory synaptic pulses at times *k* Δ, k=1,2,3,… , with Δ>0. The synaptic pulses are modeled as described in Sect. 2.4. For a given Δ>0, we determine numerically the number $N_{\Delta }$ of input pulses that are needed to generate an action potential in the target. It is important here to emphasize that the input ceases not when the target neuron actually fires, but when it is going to fire without further input pulses, in other words, when it crosses the firing threshold. Although it is hard to imagine how an actual neuronal network sending input to a target should know this number $N_{\Delta }$ without a feedback signal from the target, we can of course compute it easily in our model. Figure 6 shows the result of this computation. The maximum conductance $\bar{g}$ of an individual input pulse (see Sect. 2.4) was 10^−3^ here. We see that $N_{\Delta }$ is an increasing function of Δ, so perfect synchrony is most efficient (namely, generates firing in the target at the expense of the smallest number of input pulses).

This result is in agreement with the standard reasoning about synchronization and leakiness. For a target neuron with voltage-activated currents, such as the WB neuron, it certainly is not a priori clear that this reasoning leads to a correct conclusion. However, Fig. 6 suggests that it probably does, at least for the WB neuron.

## 4 If the Excitatory Signal Continues Until the Target Fires, Approximate Synchrony Is Optimally Efficient

Again we present several settings in which the statement in the title can be made rigorous. However, first we discuss some results concerning the firing time of a target neuron driven by a current pulse $I_{\varepsilon }$ (as in Sect. 2.1). This is useful in later subsections, and in particular it clarifies what is the essential source of the non-monotonicity found in later subsections.

### 4.1 The Time It Takes to Elicit an Action Potential with a Current Pulse

For ε>0, we denote by ${\hat{T}}_{\varepsilon }$ the time at which the action potential occurs in response to the input pulse $I_{\varepsilon }$ (as in Sect. 2.1). This definition requires several clarifications. If $I_{\varepsilon }$ elicits several action potentials, we let ${\hat{T}}_{\varepsilon }$ be the time of the earliest one. If $I_{\varepsilon }$ elicits no action potential at all, we let ${\hat{T}}_{\varepsilon }=\infty$. By “time at which the action potential occurs”, we mean the time when *v* reaches 1 for the LIF neuron, the time when *v* reaches ∞ (i.e., *θ* reaches π mod 2π) for the theta neuron, or the time when *v* rises above 0 for the WB neuron.

We also examine the ratio

$${\hat{R}}_{\varepsilon }=\frac{{\hat{T}}_{\varepsilon }}{\varepsilon },$$

which measures how long it takes to elicit an action potential *in comparison with input duration*. We note, in particular, that ${\hat{R}}_{\varepsilon }\gg 1$ implies that the input pulse is essentially over long before the target fires.

**Fig. 7:**
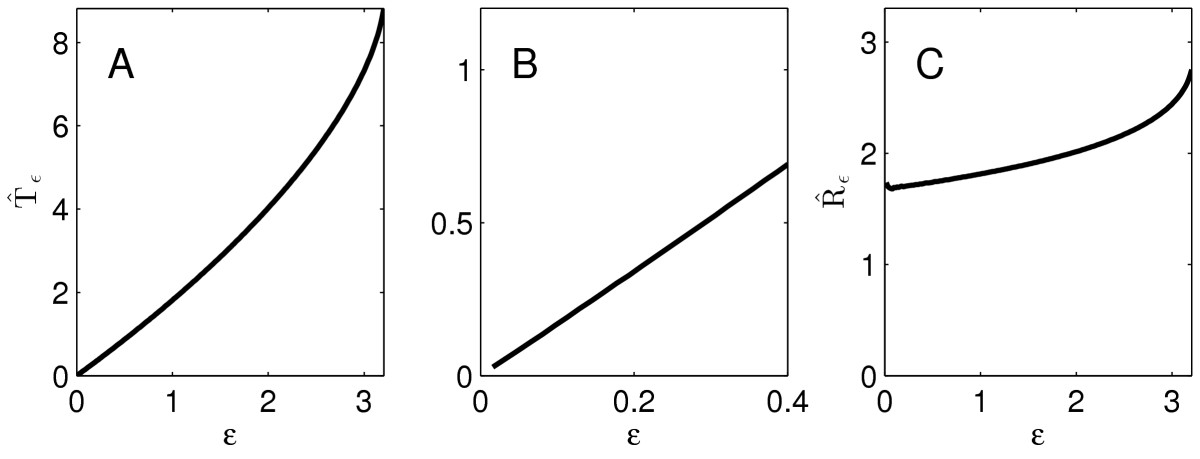
Numerical results for a LIF neuron with τ=10, starting at t=0 at the membrane potential v=0, driven by the input pulse $I_{\varepsilon }(t)=I(t/\varepsilon )/\varepsilon$, where $I(t)=2te^{-t}$. **a** The firing time ${\hat{T}}_{\varepsilon }$ as a function of *ε*. **b** Blow-up of **a** near the origin. **c** The ratio ${\hat{R}}_{\varepsilon }={\hat{T}}_{\varepsilon }/\varepsilon$ as a function of *ε*

As an example, we consider the LIF neuron with τ=10, and $I(t)=2te^{-t}$. Figure 7a shows ${\hat{T}}_{\varepsilon }$ as a function of *ε*, and Fig. 7c shows ${\hat{R}}_{\varepsilon }$ as a function of *ε*. We will prove that qualitatively, the graphs of ${\hat{T}}_{\varepsilon }$ and ${\hat{R}}_{\varepsilon }$ are always similar to those in Fig. 7 for the LIF neuron. In particular, there is no non-monotonicity here, and ${\hat{T}}_{\varepsilon }\to 0$ as ε→0, as shown in Fig. 7b, which is a blow-up of Fig. 7a.

**Theorem 3***Consider the LIF neuron given by* (6), *with*$q=\int_{0}^{\infty }I(t)dt>1$*and*$\varepsilon_{0}>0$*as described in Theorem* 1. *Then*

$$\lim_{\varepsilon \to 0}{\hat{T}}_{\varepsilon }=0\;\text{and}\;\lim_{\varepsilon \to \varepsilon_{0}}{\hat{T}}_{\varepsilon }<\infty ,$$

*and*${\hat{R}}_{\varepsilon }$*is strictly increasing for*$0<\varepsilon <\varepsilon_{0}$.

*Proof* To emphasize the dependence of ${\hat{T}}_{\varepsilon }$ on *τ*, we write ${\hat{T}}_{\varepsilon }(\tau )$. When ε=1, we write $\hat{T}(\tau )$ instead of ${\hat{T}}_{1}(\tau )$. Recall now Eq. (21):

$$v_{\varepsilon }(t,\tau )=v\left(\frac{t}{\varepsilon },\frac{\tau }{\varepsilon }\right).$$

Setting $t={\hat{T}}_{\varepsilon }(\tau )$, we find

$$1=v_{\varepsilon }\left({\hat{T}}_{\varepsilon }(\tau ),\tau \right)=v\left(\frac{{\hat{T}}_{\varepsilon }(\tau )}{\varepsilon },\frac{\tau }{\varepsilon }\right).$$

So the time at which v(t,τ/ε) becomes 1 is $t={\hat{T}}_{\varepsilon }(\tau )/\varepsilon$; but by definition that time is $\hat{T}(\tau /\varepsilon )$. We conclude

$$\hat{T}\left(\frac{\tau }{\varepsilon }\right)=\frac{{\hat{T}}_{\varepsilon }(\tau )}{\varepsilon }={\hat{R}}_{\varepsilon }(\tau ).$$

This implies that ${\hat{R}}_{\varepsilon }$ is a strictly increasing function of $\varepsilon \in (0,\varepsilon_{0})$, by Lemma 1. In the limit as ε→0, $I_{\varepsilon }(t)$ becomes qδ(t). An input pulse of the form qδ(t), with q>1, makes *v* jump above threshold instantaneously; so ${\hat{T}}_{\varepsilon }\to 0$ as ε→0. The limit of ${\hat{T}}_{\varepsilon }$ as $\varepsilon \to \varepsilon_{0}$ is the finite time at which $v_{\varepsilon_{0}}$ reaches 1. □

**Fig. 8:**
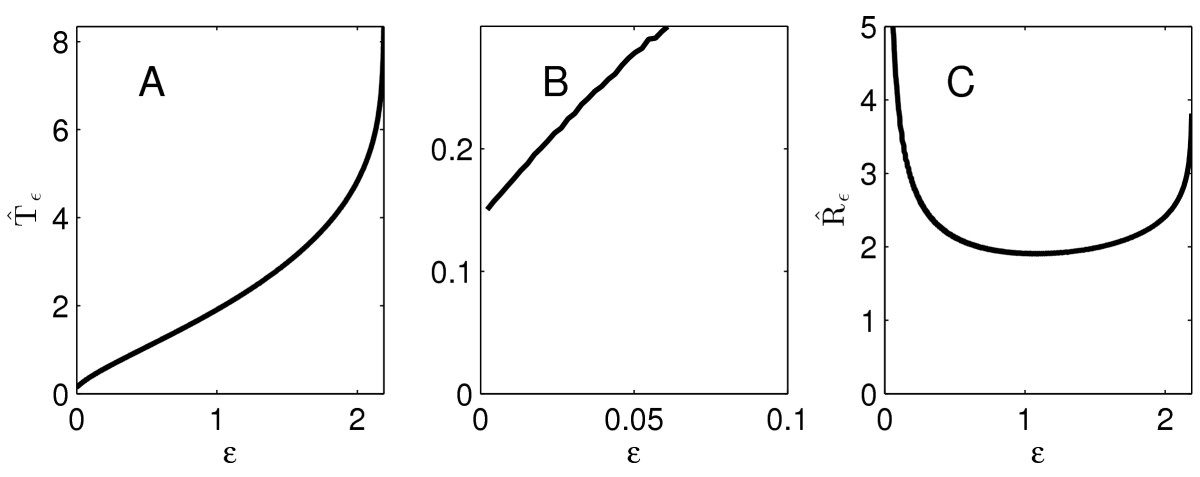
Like Fig. 7, for the theta neuron with τ=1/2, $I(t)=4te^{-t}$

That fact that ${\hat{T}}_{\varepsilon }\to 0$ as ε→0 in Fig. 7a is a bit unrealistic from a biological point of view. In a real neuron, an instantaneous charge injection (if there were such a thing in reality) would have to be of gigantic strength to push the membrane potential above 0 mV instantly, and thereby—by our definitions—trigger an instant action potential. We next give numerical results for the theta neuron with τ=1/2 and $I(t)=4te^{-t}$. Figures 8a and 8c show ${\hat{T}}_{\varepsilon }$ and ${\hat{R}}_{\varepsilon }$ as functions of *ε*; Fig. 8b is a blow-up of Fig. 8a near ε=0. We will show that, for the theta neuron, the graphs of ${\hat{T}}_{\varepsilon }$ and ${\hat{R}}_{\varepsilon }$ always share important features of the examples shown in Fig. 8. In particular, the limit of ${\hat{T}}_{\varepsilon }$ as ε→0 is positive, and ${\hat{R}}_{\varepsilon }$ depends on *ε* non-monotonically.

**Theorem 4***For the theta neuron with a positive input pulse*$I_{\varepsilon }$, *with*q>1*and*$\varepsilon_{0}>0$*defined as described in Theorem* 2, *the firing time*${\hat{T}}_{\varepsilon }$*satisfies*

24 $$\lim_{\varepsilon \to 0}{\hat{T}}_{\varepsilon }>0\;\text{and}\;\lim_{\varepsilon \to \varepsilon_{0}}{\hat{T}}_{\varepsilon }=\infty .$$

*The function*${\hat{R}}_{\varepsilon }={\hat{T}}_{\varepsilon }/\varepsilon$*is non*-*monotonic*, *with*

25 $$\lim_{\varepsilon \to 0}{\hat{R}}_{\varepsilon }=\lim_{\varepsilon \to \varepsilon_{0}}{\hat{R}}_{\varepsilon }=\infty .$$

*Proof* In the limit as ε→0, $I_{\varepsilon }$ becomes qδ(t), and ${\hat{T}}_{\varepsilon }$ therefore converges to the positive, finite time that it takes for the solution of

$$\frac{dv}{dt}=-\frac{v}{\tau }(1-v)$$

to rise from q>1 to ∞. This proves $\lim_{\varepsilon \to 0}{\hat{T}}_{\varepsilon }>0$. Because $\lim_{t\to \infty }v_{\varepsilon_{0}}(t)=1$ (see discussion at the end of Sect. 3.3, and in particular Fig. 5), $\lim_{\varepsilon \to \varepsilon_{0}}{\hat{T}}_{\varepsilon }=\infty$ follows from the continuous dependence of $v_{\varepsilon }$ on *ε*. Finally, (25) follows immediately from (24). □

**Fig. 9:**
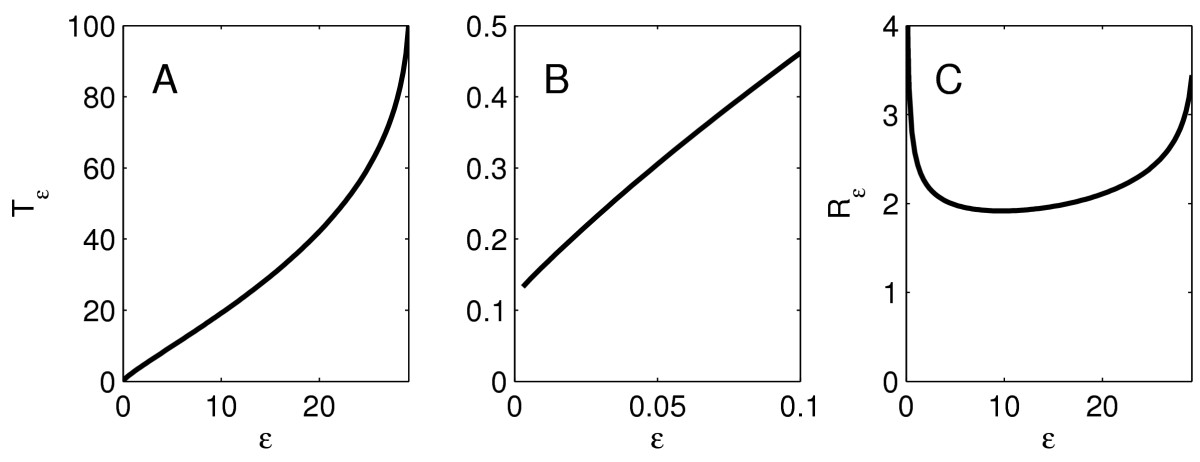
Like Fig. 8, for the WB neuron, $I(t)=20te^{-t}$

Next we present numerical simulations suggesting that the behavior of ${\hat{T}}_{\varepsilon }$ and ${\hat{R}}_{\varepsilon }$ for the WB neuron is similar to that for the theta neuron. For illustration, we consider the example $I(t)=20te^{-t}$. We start, at time t=0, with *v*, *h*, and *n* at the equilibrium values corresponding to zero external drive. Figures 9a and 9c show ${\hat{T}}_{\varepsilon }$ and ${\hat{R}}_{\varepsilon }$ as functions of *ε*; Fig. 9b is a blow-up of Fig. 9a near ε=0.

### 4.2 If Input Current Ceases when the Target Fires, How Much Charge Is Injected?

The integral

$$Q_{\varepsilon }=\int_{0}^{{\hat{T}}_{\varepsilon }}I_{\varepsilon }(t)dt$$

is the total amount of charge needed to elicit the action potential. We note that

$$\int_{0}^{{\hat{T}}_{\varepsilon }}I_{\varepsilon }(t)dt=\int_{0}^{{\hat{T}}_{\varepsilon }}\frac{1}{\varepsilon }I\left(\frac{t}{\varepsilon }\right)dt=\int_{0}^{{\hat{T}}_{\varepsilon }/\varepsilon }I(s)ds=\int_{0}^{{\hat{R}}_{\varepsilon }}I(s)ds,$$

so

26 $$Q_{\varepsilon }=\int_{0}^{{\hat{R}}_{\varepsilon }}I(s)ds.$$

**Fig. 10:**
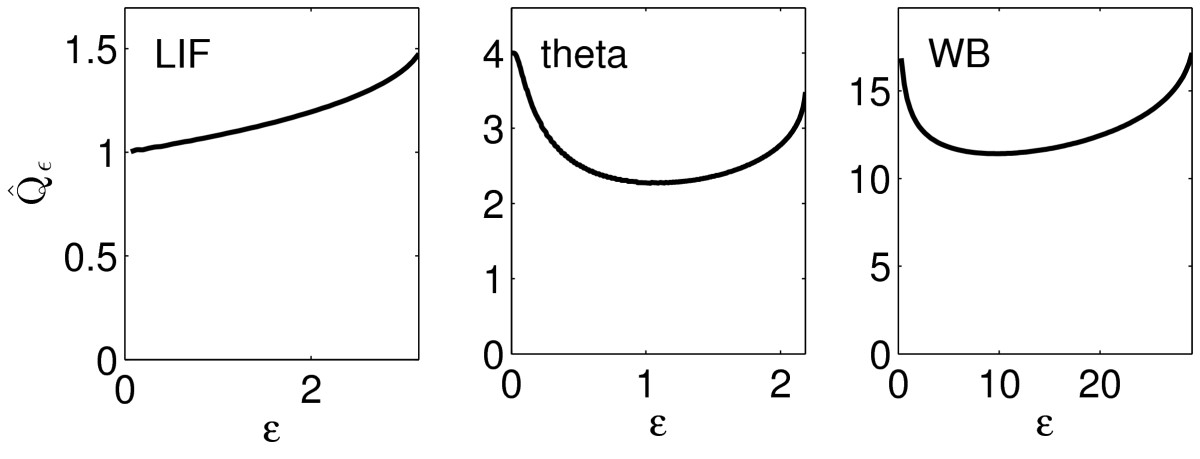
${\hat{Q}}_{\varepsilon }$ as a function of *ε*, for the examples of Figs. 7, 8, and 9

Thus ${\hat{Q}}_{\varepsilon }$ depends on *ε* monotonically if and only if ${\hat{R}}_{\varepsilon }$ does. For the LIF, theta, and WB neurons, we show in Fig. 10 the dependence of ${\hat{Q}}_{\varepsilon }$ on *ε*. In accordance with the preceding reasoning and with the results of Sect. 4.1, ${\hat{Q}}_{\varepsilon }$ increases with *ε* for the LIF neuron, but not for the QIF and WB neurons.

**Fig. 11:**
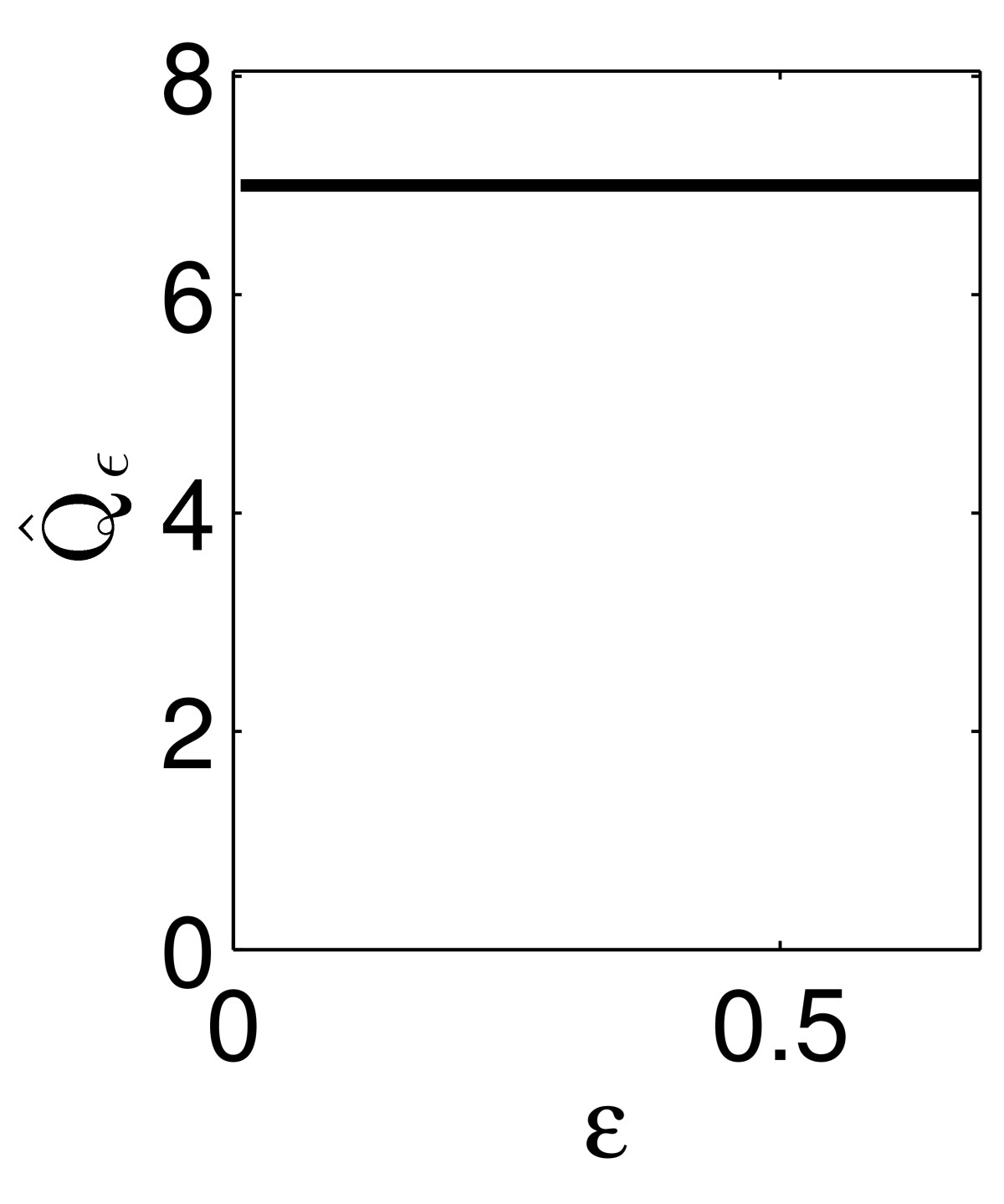
${\hat{Q}}_{\varepsilon }$ as a function of *ε*, for the example of Fig. 9, with $I(t)=7te^{-t}$. For values of *ε* outside the window shown, the input $I_{\varepsilon }$ does not trigger an action potential

The variation of ${\hat{Q}}_{\varepsilon }$ as a function of *ε* is relevant only if ${\hat{R}}_{\varepsilon }$ is not large, i.e., if the input pulse is so strong that the target fires while the input is still ongoing. When ${\hat{R}}_{\varepsilon }\gg 1$, then the input pulse is essentially complete by the time the target fires, and therefore ${\hat{Q}}_{\varepsilon }$ is simply (very close to) *q*. To illustrate this, we show in Fig. 11 the same figure as in the right-most panel of Fig. 10, but with $I(t)=20te^{-t}$ replaced by $I(t)=7te^{-t}$. Here $\min_{\varepsilon }{\hat{R}}_{\varepsilon }\approx 50$, and ${\hat{Q}}_{\varepsilon }$ is very close to independent of *ε*.

### 4.3 If Synaptic Input Pulses Cease when the Target Fires, How Many Pulses Are Needed?

**Fig. 12:**
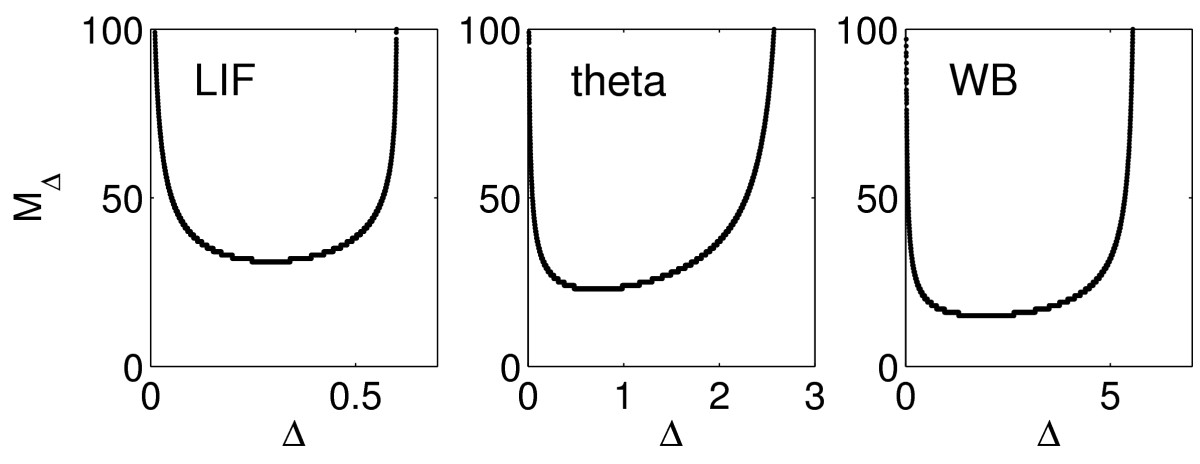
Number of input pulses that have arrived by the time the target fires, for the LIF, theta, and WB models, with $\bar{g}=0.005$, as a function of Δ=time between input pulses

Arguably, this is the version of our question that is biologically most interesting. We consider trains of weak excitatory synaptic inputs as described in Sect. 2.4. Recall, in particular, from Sect. 2.4 that we denote by Δ>0 the time between input arrivals. We denote by $M_{\Delta }$ the number of input pulses that will arrive before the target fires. We emphasize that $M_{\Delta }$ is not the same as the “$N_{\Delta }$” of Sect. 3.5; $N_{\Delta }$ is the number of input pulses needed to take the target above the firing threshold, while $M_{\Delta }$ is the number of input pulses that will have arrived by the time the target actually fires. Figure 12 shows $M_{\Delta }$ as a function of Δ, for the LIF, theta (QIF), and WB models, using $\bar{g}=0.005$. The figure confirms the insight from Fig. 1: When the input pulses are less synchronous (that is, when Δ is larger), fewer of them may have to arrive before the target fires. The figure shows that this effect can be quite significant.

### 4.4 Linearly Rising Current Input Driving a LIF Neuron

**Fig. 13:**
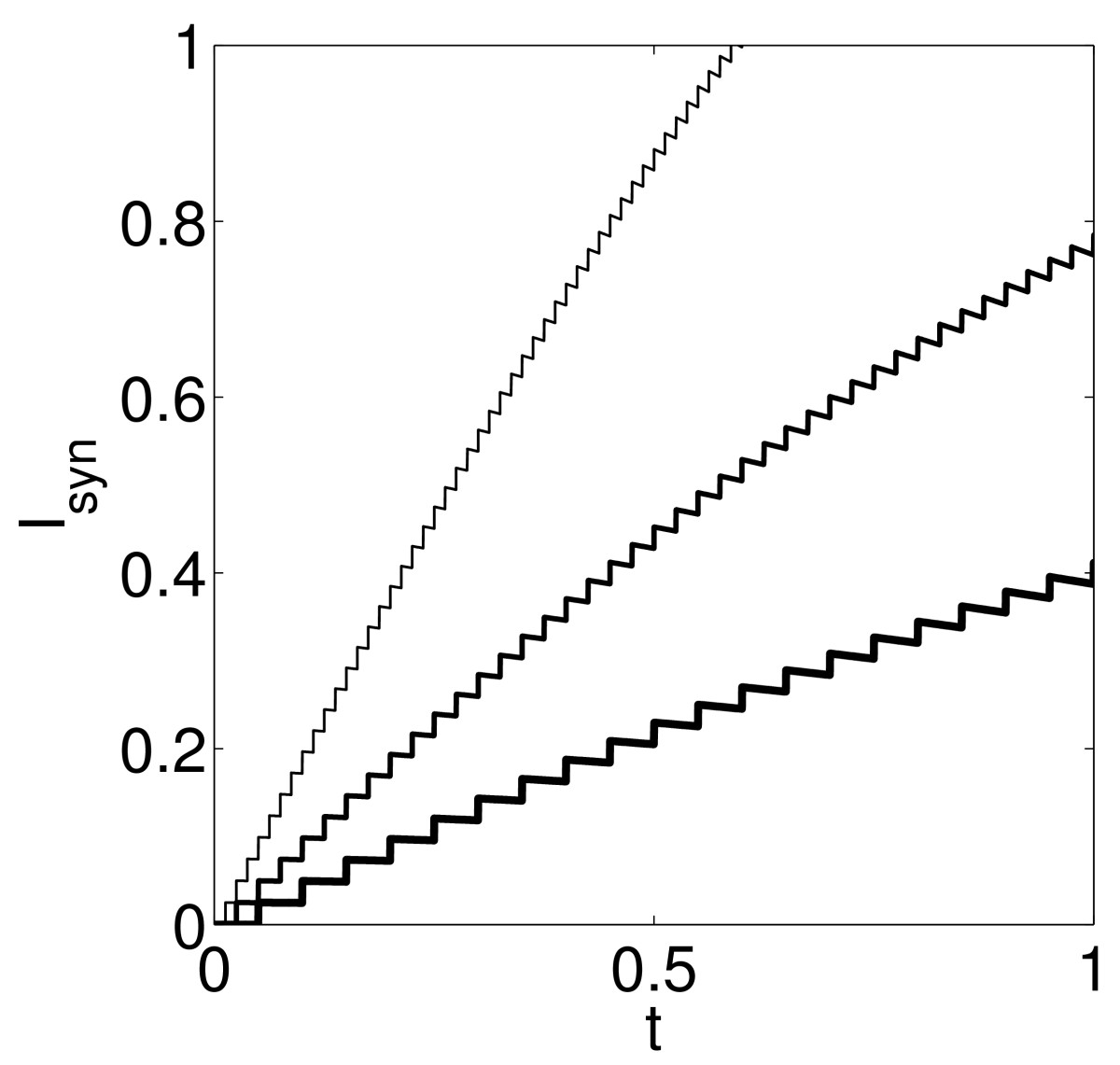
Synaptic input current into the LIF neuron of Fig. 12, with Δ=0.05, 0.025, and 0.0125. (The smaller Δ, the steeper is the *curve*)

When Δ, the time between input pulses, is small in comparison with the synaptic decay time constant, taken to be 3 ms here (see Eq. (17)), the input currents in Fig. 12 rise approximately linearly, since the synaptic gating variable builds up with each input pulse, and it decays only little between pulses. Figure 13 illustrates this, by plotting, for three of the simulations underlying the left panel of Fig. 12, namely the ones for Δ=0.05, 0.025, and 0.0125, the current $I_{\mathrm{syn}}=\bar{g}s(t)(5-v)$.

**Fig. 14:**
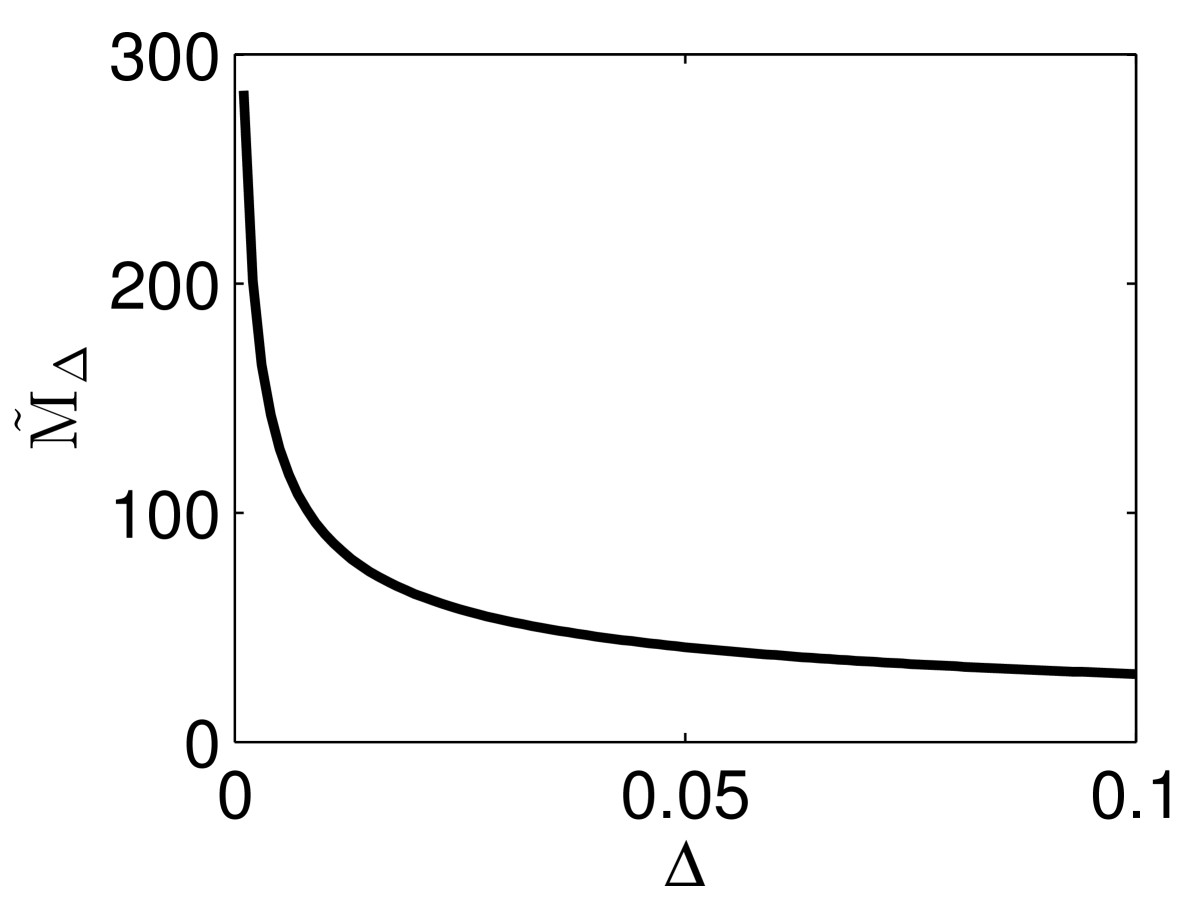
${\tilde{M}}_{\Delta }$ as a function of Δ, computed from the simplified model problem (27). This model problem only captures the range in which $M_{\Delta }$ (approximated here by ${\tilde{M}}_{\Delta }$) is a decreasing function of Δ

The slope of the linear build-up of $I_{\mathrm{syn}}$ is approximately proportional to 1/Δ. We therefore think about the following model problem:

27 $$\frac{dv}{dt}=-\frac{v}{\tau }+\frac{c}{\Delta }t,\;v(0)=0,$$

where c>0. We compute the time ${\tilde{T}}_{\Delta }$ at which *v* reaches 1, then define ${\tilde{M}}_{\Delta }$ to be the number of pulses arriving in time ${\tilde{T}}_{\Delta }$, i.e.,

28 $${\tilde{M}}_{\Delta }=\frac{{\tilde{T}}_{\Delta }}{\Delta },$$

and plot ${\tilde{M}}_{\Delta }$ as a function of Δ. For τ=10, c=0.025, the result is shown in Fig. 14. We see that the model problem (27) captures the central fact that $M_{\Delta }$ (approximated here by ${\tilde{M}}_{\Delta }$) is a decreasing function of Δ for small Δ (and thus perfect synchrony is not optimal), but not the fact that it is an increasing function of Δ for large Δ (see Fig. 12). This is not surprising: In the left-most panel of Fig. 12, in the range when $M_{\Delta }$ is an increasing function of Δ, the duration of the input spike volley, $M_{\Delta }\Delta$, is much greater than 3, the decay time constant of the excitatory synaptic input pulses. In this regime, the assumption of a linearly building input current is not valid.

In spite of the simplicity of (27), it is not possible to write down a formula for ${\tilde{T}}_{\Delta }$. To see this, note first that the solution of (27) is

$$v(t)=\frac{c}{\Delta }(t-\tau )\tau +\frac{c}{\Delta }\tau^{2}e^{-t/\tau }.$$

$${\tilde{T}}_{\Delta }$$

solves the equation $v({\tilde{T}}_{\Delta })=1$, i.e.,

29 $$e^{-{\tilde{T}}_{\Delta }/\tau }-\left(1-\frac{{\tilde{T}}_{\Delta }}{\tau }\right)=\frac{\Delta }{c\tau^{2}}.$$

One cannot solve this equation explicitly, but for small-enough Δ, ${\tilde{T}}_{\Delta }\ll \tau$, and (29) is then well approximated by

$$\frac{1}{2}\frac{{\tilde{T}}_{\Delta }^{2}}{\tau^{2}}=\frac{\Delta }{c\tau^{2}},$$

i.e., ${\tilde{T}}_{\Delta }=\sqrt{2\Delta /c}$, and ${\tilde{M}}_{\Delta }=\sqrt{2/(c\Delta )}$. It is easy to argue that this approximate calculation rigorously describes the asymptotic behavior of ${\tilde{M}}_{\Delta }$, calculated from the model problem (27), as Δ→0.

**Theorem 5***The quantity*${\tilde{M}}_{\Delta }$, *defined in* (28) *based on the model problem* (27), *satisfies the asymptotic relation*

$${\tilde{M}}_{\Delta }∼\sqrt{\frac{2}{c}}\Delta^{-1/2}$$

*in the limit as*Δ→0.

In fact, the blow-up in the limit as Δ→0 in Fig. 12 can be verified numerically to be proportional to $\Delta^{-1/2}$ as well, in all three cases shown in the figure.

## 5 Discussion

We return to Fig. 1. The figure shows that with greater tonic inhibition of the E-cells (right panel), the degree of synchrony among the E-cells is reduced, yet the number of participating E-cells is reduced as well. In the notation that we have used throughout this article, for larger Δ (right panel), the number of input pulses to the I-cells required to elicit firing is smaller. Section 4 explains how this comes about.

In general, in PING, the E-cells synchronize approximately, but not perfectly, when different E-cells receive different drives, or there is heterogeneity in synaptic strengths. The I-cells are therefore driven to firing by a sequence of nearly, but not perfectly synchronous input pulses. Our results show that there is an optimal level of looseness in the synchronization of the E-cells, that is, a level of looseness that allows operating the PING rhythm with the minimal number of E-cell action potentials.

In reality, when a feedback signal terminates the input, that feedback signal would not likely come at the moment when the target fires. A delay in the feedback signal amplifies our point: During the delay time, input is “wasted”, and the more synchronous the input stream, the more input is wasted.

We have concluded that perfect synchrony is optimal if the input stream is allowed to have the “foresight” of ceasing when the firing threshold is reached in the target. Note, however, that there is no well-defined “time at which the firing threshold is reached” when the target is not a single neuron, but a heterogeneous network. We therefore hypothesize that of the two principles stated in the Introduction, the second is the more relevant from the point of view of biology.

An interesting question for future study is how noise affects our conclusions. The answer depends almost certainly on how the question is made precise. The simplest formalization of the question, in the LIF framework, might be as follows. Consider

$$dv_{\varepsilon }=-\frac{v_{\varepsilon }}{\tau }dt+\sigma dW+I_{\varepsilon }(t),\;t\geq 0,$$

where *dW* denotes normalized Gaussian white noise, so that dv=−(v/τ)dt+σdW, without the extra input pulse $I_{\varepsilon }$, would be an Ornstein–Uhlenbeck process. Assume that $v_{\varepsilon }(0)$ has Gaussian distribution with mean 0 and variance $\sigma^{2}\tau /2$, the equilibrium distribution of the Ornstein–Uhlenbeck process. Define $F(\varepsilon )=P(\sup_{0\leq t<c\varepsilon }v_{\varepsilon }(t)>1)$, where c>0 is of moderate size, perhaps c=3. If *F* is a strictly decreasing function of *ε*, then synchrony is, in this sense, “optimal”, whereas it isn’t if *F* has a local maximum at a positive value of *ε*. More realistic variations on this formalization are of course possible, using noise-driven Hodgkin–Huxley-like neurons with conductance-based inputs. We would not be surprised if *F* turned out to be strictly decreasing, i.e., perfect synchrony turned out to be “optimal” in this sense, but will leave the study of this issue to future work.

We summarize our surprising conclusion: The commonplace and widely accepted argument suggesting that synchrony makes excitatory inputs more effective is, at least in one very natural formalization (namely, that of Sect. 4), wrong. It is not just “slightly wrong”, but significantly so; see for instance the right-most panel in Fig. 10, which shows that, for the WB neuron, the “optimal” (in the sense of Sect. 4) duration of an input spike volley is on the order of 10 ms.

## Authors’ original submitted files for images

Below are the links to the authors’ original submitted files for images. Authors’ original file for figure 1 Authors’ original file for figure 2 Authors’ original file for figure 3 Authors’ original file for figure 4 Authors’ original file for figure 5 Authors’ original file for figure 6 Authors’ original file for figure 7 Authors’ original file for figure 8 Authors’ original file for figure 9 Authors’ original file for figure 10 Authors’ original file for figure 11 Authors’ original file for figure 12 Authors’ original file for figure 13 Authors’ original file for figure 14
